# Computer simulation of scavenging by hominins and giant hyenas in the late Early Pleistocene

**DOI:** 10.1038/s41598-023-39776-1

**Published:** 2023-09-28

**Authors:** Jesús Rodríguez, Ericson Hölzchen, Ana Isabel Caso-Alonso, Jan Ole Berndt, Christine Hertler, Ingo J. Timm, Ana Mateos

**Affiliations:** 1 National Research Center On Human Evolution (CENIEH), Paseo Sierra de Atapuerca 3, 09002 Burgos, Spain; 2 Chair for Business Informatics 1, Trier University, Behringstraße 21, 54296 Trier, Germany; 3 German Research Center for Artificial Intelligence (DFKI). Smart Data and Knowledge Services - Cognitive Social Simulation, Trier University, Behringstraße 21, 54296 Trier, Germany; 4 Facultad de Ciencias. Edificio de Biología, Universidad Autónoma de Madrid. C/ Darwin, 2. Campus de Cantoblanco, 28049 Madrid, Spain; 5 The Role of Culture in Early Expansion of Humans (ROCEEH), Senckenberg Research Institute, Senckenberganlage 25, 60325 Frankfurt Am Main, Germany; 6 The Role of Culture in Early Expansion of Humans (ROCEEH), Heidelberg Academy of Sciences, Karlstraße 4, 69117 Heidelberg, Germany

**Keywords:** Ecological modelling, Palaeoecology, Archaeology

## Abstract

Consumption of animal-sourced food is an important factor in broadening the diet of early hominins, promoting brain and body growth, and increasing behavioural complexity. However, whether early hominins obtained animal food by scavenging or hunting large mammals remains debated. Sabre-toothed felids have been proposed to facilitate the expansion of early *Homo* out of Africa into Europe 1.4–0.8 Ma by creating a niche for scavengers in Eurasia as the carcasses abandoned by these felids still contained abundant edible resources. In contrast, it has been argued that the niche for a large scavenger was already occupied in Eurasia by the giant hyena, preventing hominins from utilising this resource. This study shows that sabre-toothed felids generated carcasses rich in edible resources and that hominins were capable of competing with giant hyenas for this resource. The simulation experiments showed that maintaining an optimum group size is essential for the success of the hominin scavenging strategy. Early hominins could outcompete giant hyenas only if they could successfully dispute carcasses with them. Thus, in the presence of a strong competitor, passive scavenging is essentially the same as confrontational scavenging.

## Introduction

Hominins arrived to southern Europe at least 1.4 Ma ago^1–4^ and were settled there during the Epivillafranchian (approximately 1.2–0.8 Ma)^5^. However, the roles of changing climate, palaeogeography, faunal assemblages, and other environmental drivers in their dispersion into Europe are under debate^6–9^. A key question is how the large European mammalian fauna, especially the composition of the carnivore guild, influenced the accessibility of the early hominins to animal food resources^10–13^. Although the dichotomy of hunting vs. scavenging as the main foraging strategies of early *Homo* is still unresolved^14–23^, scavenging has been a common adaptive behaviour in the genus *Homo* since its origins^24,25^. Thus, despite the fact that the first hominins in Europe were likely omnivores^8^, it may be assumed that scavenging was part of their behavioural repertoire (Supplementary Note S1).

The scavenging opportunities for a hominin species in a particular ecological scenario can be determined by the complex interaction of several factors, such as the density, size, and quality of carcasses dispersed around the landscape, which can further be determined by the abundance, behavioural, and morphofunctional characteristics of the predators and by the ecological characteristics and abundance of their potential prey^8,26^. The other main factors to be considered are the presence of competitors and their ecological characteristics and behaviours. It has been suggested that sabre-toothed felids generated many large carcasses because of their inability to entirely consume their kills^27,28^, facilitating the survival of early *Homo* during the Epivillafranchian (Supplementary Note S1). This argument is usually applied to species of the genus *Megantereon*, but may also be applied to *Homotherium* if solitary behaviour is assumed. Nevertheless, quantitative estimates of the rate of carcass production by these predators and of the amount of nutrients in the abandoned carcasses are currently lacking. In this scenario, an opposite but equally important role, was played by the giant hyena (*Pachycrocuta brevirostris*)^12,29,30^, frequently regarded as a “hyperscavenger” and direct competitor of hominins^31^. Indeed, it has been claimed that the Fuente Nueva-3 site provides evidence of the direct competition between hominins and giant hyenas for an elephant carcass^32^. Moreover, it has been suggested that the giant hyena was dependent on the partially consumed carcasses produced by sabre-toothed cats, such that the decline of *Pachycrocuta* in Europe was linked to the extinction of sabre-toothed cats, particularly *Megantereon whitei*^29,30^.

If hominins practised a flexible strategy of carrion acquisition, several foraging scenarios are possible^15,16,18–20,33^. It has been proposed that groups of hominins were capable of stealing the kills of large predators (confrontational scavenging or kleptoparasitism)^17^. Moreover, endurance running was suggested to be an advantage for hominins when competing with giant hyenas for carrion, as discussed in^4^. However, the real advantages provided by endurance running is a controversial topic^34^. An alternative strategy would be the passive scavenging of partially or completely defleshed carcasses and interference competition for carrion with giant hyena^12,30,35^. Group size was likely a key factor for both strategies, as a group of hominins was large enough to defend a carcass from any direct competitor.

In this study, quantitative estimates of the nutrients contained in the carcasses abandoned by the main Epivillafranchian large predators are provided. Moreover, the competition for carrion between hominins and giant hyenas is approached here through the energetic costs and returns that this interaction represents for both species. Computer-based simulation experiments were performed to simulate the competition between hominins and giant hyenas, evaluate the feasibility of passive scavenging by hominins, and determine the factors that influenced scavenging in the Epivillafranchian ecosystems of the Iberian Peninsula. We aimed to evaluate the effect of ecosystem carrying capacity on the feasibility of passive scavenging and how the size of the hominin group affected the efficiency of this strategy. Although the simulations are an oversimplification of the trophic niche of hominins, this is a necessary methodology for understanding and examining the competition among hominins and hyenas in a tractable and efficient way^36^.

## Results

The effects of hominin group size and predator density on the competition for carrion between hominins and giant hyenas in two different ecological scenarios were evaluated using six simulation experiments (Table 1). Each experiment was replicated 70 times (see Methods and Supplementary Methods S1), and all the outputs of the 420 replicates (70 runs × six experiments) are shown in Supplementary Dataset 1. For the experiments, it was assumed that giant hyenas were strict solitary scavengers and hominins competed with them for the carcasses produced by large felids (*Homotherium latidens*, *Megantereon whitei,* and *Panthera gombaszoegensis*) in a trophic strategy of passive scavenging.

**Table 1:** Summary of the six simulation experiments performed to test the effect of hominin group size and predator density on the competition for carrion between hominins and giant hyenas during the Epivillafranchian. The experiments were conducted in two ecological scenarios, differentiated by the composition of the large carnivore guild. The experiments evaluated the effect of varying the size of the hominin bands from 1 to 25 individuals in steps of three individuals. Each experiment was replicated 70 times as determined by using the precision analysis (Supplementary Methods S1).

		Hominin group size	*Homotherium* (Cats/100 km^2^)	*Megantereon* (Cats/100 km^2^)	*Panthera* (Cats/100 km^2^)
Scenario 1	Experiment 1	1, 4, 7, 10, 13, 16, 19, 22, 25	2	5	3
	Experiment 2		3.5	8.7	5.4
	Experiment 3		6	15	9
Scenario 2	Experiment 4		2	5	0
	Experiment 5		3.5	8.7	0
	Experiment 6		6	15	0

### Carrion production in the Epivillafranchian

Estimated amounts of energy available in the carcasses abandoned by the three large predators were obtained based on the body weight of their preferred prey, the estimated hunting frequency, and the daily intake rate of the predators, as detailed in the “Methods” section. The values included in the experiments are listed in Table 2. These estimates are in general agreement with the values observed in recent ecosystems (Supplementary Methods S2) and confirm the assertion that the two sabre-toothed species generated far more scavengeable resources than recent and fossil pantherines.

**Table 2:** Edible resources in the carcasses abandoned by the large carnivores considered in the experiments. The body mass of the preferred prey is based on isotopic analyses of fossils from Venta Micena^80^. The wastage factor accounts for the non-edible percentage of a carcass and is dependent on the body mass^82^.

		*Homotherium*	*Megantereon*	*Panthera*
V_1_	Body weight (kg)^85^	200	100	105
V_2_	Interval between kills (days)	8	7	7
V_3_	Kill rate (prey/h) V_3_ = V_2_/24	0.0052	0.006	0.006
V_4_	Preferred prey body mass (kg)	400	350	200
V_5_	Wastage factor	0.35	0.35	0.3
V_6_	Caloric return per kill (kcal) V_6_ = V_4_*V_5_*1300	182,000	159,250	78,000
V_7_	Absolute intake rate (kcal/day)^81^ Log (V_7_) = 0.28 + 0.70 Log (1000 V_1_)	9365.8	5777.9	5977.7
V_8_	Energy consumption per kill (kcal) V_8_ = V_7_*V_3_	74,927	40,445	41,844
V_9_	Energy left on carrion (kcal) V_9_ = V_6_–V_8_	107,073	118,805	36,156

**Figure 1:**
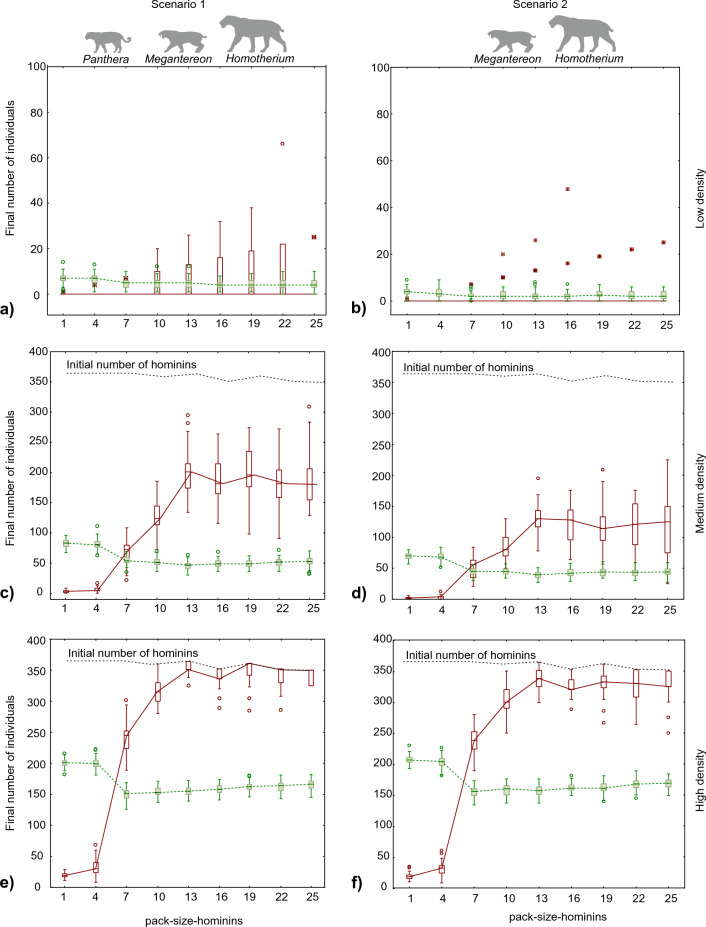
Final number of scavengers in the experiments. Hominins are represented in red (continuous line) and hyenas in green (dashed line). Three experiments were performed by varying the densities of predators for Scenario 1 (**a**, **c**, **e**) and Scenario 2 (**b**, **d**, **f**) from low density (**a**, **b**) to medium density (**c**, **d**) to high density (**e**, **f**). The limits of the boxes correspond to the first and third quartiles; the median is presented with a horizontal line. The whiskers mark the maximum and minimum without outliers or extreme values. Outliers and extreme values are indicated with a white dot and an asterisk, respectively.

The results for the two scenarios were similar. As expected, the final populations of both hyenas and hominins were larger in experiments with higher predator densities (Fig. 1). Indeed, the main difference between the results of both scenarios was that the available resources were insufficient to sustain a hominin population when the predator density was low and only two sabre-toothed cats were present (Fig. 1b). In the scenario of low resources, only giant hyenas survived, although at a low population density (Fig. 1b). In contrast, the presence of a third predator (*P. gombaszoegensis*) increased the available carrion enough to sustain the populations of both scavengers in most runs, even at a low predator density (Fig. 1a).

### Competition between hominins and giant hyenas

Hominin group size (hominin-pack-size) can predict the competition between hominins and giant hyenas. The final number of hyenas exceeded that of hominins when the size of the hominin group was less than five (Fig. 1), which was arbitrarily set as the threshold necessary for a hominin group to chase away a single hyena. Moreover, hominins could not survive until the end of the simulations when their group size was less than five and the population density of predators was low or medium. When the hominin groups were larger than five, the final population of hominins was larger than that of giant hyenas; however, giant hyenas subsisted under all the conditions tested. The positive effect of increasing hominin group size on the final population of hominins continued until a group size of 13 individuals was reached and levelled off beyond this point (Fig. 1). This pattern can be explained by the fact that a group of more than 10 hominins was necessary to chase away any predator. The differences in the final number of hominins for groups of 13 or more individuals were due to variations in the initial number of groups at the start of the simulation (Fig. 1). This variation was caused by the rounding down of the number of groups to a closer integer after dividing the initial population by the established hominin-pack-size.

**Figure 2:**
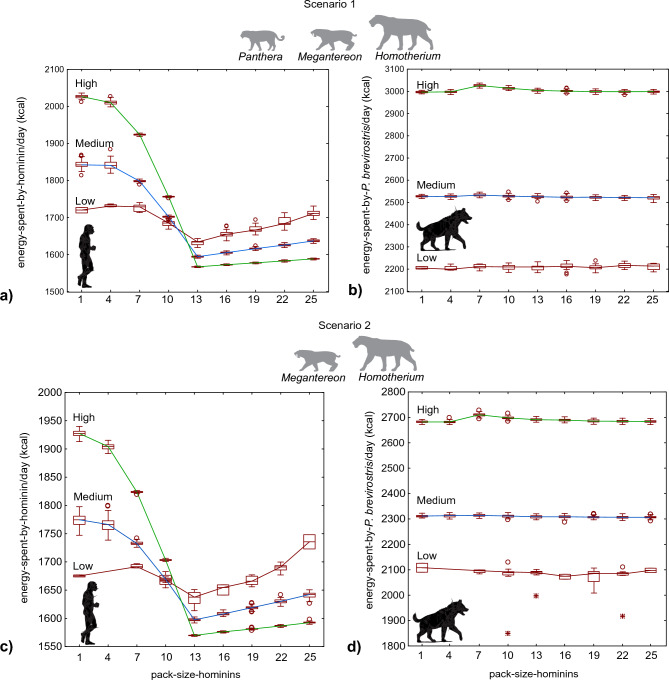
Energy expenditure of scavengers. The results of the experiments are shown in relation to the size of the hominin groups. Three experiments were performed for each scenario by varying the densities of predators from low density (red line) to medium density (blue line) to high density (green line). The limits of the boxes correspond to the first and third quartiles; the median is shown by a horizontal line. The whiskers mark the maximum and minimum without outliers and extreme values. Outliers and extreme values are indicated with a white dot and an asterisk, respectively.

Energy expenditure is simulated in SCAVCOMP-ABM by letting agents to expend energy at their basal metabolic rate when quiet and at a higher rate when moving (see Methods). Individual hominin energy expenditure decreased with hominin group size, whereas hyena energy expenditure was mostly unaffected by hominin group size (Fig. 2). The effect was difficult to detect at a low predator density in Scenario 2 (Fig. 2c) because the hominins survived to the end of the simulation in very few runs, but it was clearer in Scenario 1 (Fig. 2a). Nevertheless, the pattern appeared to be the same at all three levels of predator density assessed in the experiments. Energy expenditure per hominin decreased steeply with group size until the group size was 13 and increased gently beyond this point. This suggests the existence of an optimal group size that reduces the energy investment required for an activity. The optimum group size was found to be related to the strength necessary to chase away predators and competitors. The decrease in energy expenditure with respect to group size was also influenced by predator density. The higher the predator density, the greater the difference in energy expenditure between individuals in large and small groups of hominins. Similarly, the energy expenditure of hyenas was higher when the predator density was high, since the number of interactions with predators increased (Fig. 2b,d).

## Discussion

Scavenging is a fundamental factor in the structure of carnivorous communities in terrestrial ecosystems^37^. Moreover, the role of scavenging in the evolution and expansion of early hominins is a frequently debated and controversial issue^19,20,22^. The simulation experiments suggest that passive scavenging could be a very successful strategy for late-early Pleistocene hominins in Europe, even in competition with giant hyenas. Only when hominins foraged in very small groups, the ecosystem productivity was low, and the population densities of *Megantereon* and *Homotherium* were low or moderate*,* giant hyenas displaced the hominins. However, our simulations considered scavenging a unique procurement strategy for hominins, and this is an entirely unrealistic assumption. The hominins may be assumed to exhibit flexible omnivorous behaviour and are capable of adopting their diet by exploiting different plant and animal resources, including carrion, according to their availability^6,8,38,39^.

A requisite for the coexistence of two large scavengers, hyenas and hominins, is the availability of sufficient carcasses containing large amounts of edible resources. It has been suggested that these resources existed in Early Pleistocene Europe, owing to the presence of two sabre-tooths, especially *Megantereon*^29,35^. The estimates of the number of edible resources on the carcasses of large ungulates abandoned by the two sabre-toothed species support the interpretation of sabre-tooths as significant carrion providers. If *Megantereon* killed one prey every week, based on a conservative estimate, only one-third of the edible energy in the carcass would be consumed before killing a new prey (Table 2). This estimate supports the claims linking the extinction of the giant hyena in Europe to the extinction of *Megantereon*^29,30^. Interestingly, although this argument is usually applied only to *Megantereon,* our estimations suggest that *Homotherium* also produces carcasses with similar amounts of edible resources. In contrast, the resources contained in the carcasses of prey killed by the European jaguar would be markedly lower (Table 2) and similar to the average caloric content of the carcasses abandoned by recent large predators^40^. However, it should be acknowledged that the role of *Homotherium* as a producer of carcasses with high nutrient content relies on the assumption that it was a solitary species. If *Homotherium* was a social felid, as sometimes suggested^41^, a pack would be able to consume a large proportion of the carcass before abandoning it. Indeed, according to the estimates in Table 2, a pack of five *Homotherium* individuals would require approximately 45,000 kcal/day and consume the entire carcass of a 400 kg ungulate every 3–4 days. In such cases, only some portions, such as the brain and bone marrow, remain in the carcass because of the sabre-tooth’s inability to break bones^41^.

Our simulations modelled *Pachycrocuta brevirostris* as a solitary passive scavenger; however, this decision may be controversial because it has been proposed that *P. brevirostris* occupied a niche similar to that of extant spotted hyenas (*Crocuta crocuta*), which are highly active hunters and kleptoparasites^29^. However, the taphonomic study of the bone assemblage preserved at Venta Micena^27,30,42^ together with the morphofunctional analyses of the mandibles and teeth of *Pachycrocuta* from several European localities^30^, strongly suggests that the giant hyena was a dedicated strict scavenger or specialised kleptoparasite that stole the prey of sabre-tooths and other large carnivores. A possible argument against considering *Pachycrocuta* a passive scavenger^30^ is that carrion eaters must range over large areas in search of food, a task to which the large and non-cursorial giant hyena is not especially adapted. Based on this argument, giant hyenas did not prospect the environment in search of carrion but pursued other predators and stole their preys^30^. Thus, giant hyenas were kleptoparasites rather than passive scavengers. In this regard, their behaviour would be similar to that of recent spotted hyenas, as suggested by Turner and Antón^29^. Nevertheless, the simulation experiments suggest that if the carrion is sufficiently abundant, high mobility is not required for a passive scavenger. The walking speed of *P. brevirostris* was set at 5 km/h (the same as that of the hominins), and it was assigned a high energy expenditure during movement. Moreover, if giant hyenas were kleptoparasites, they would have frequent primary access to carcasses, and the bone accumulation generated by giant hyenas would be difficult to differentiate from that generated by hunting carnivores.

The social behaviour of giant hyenas is another potentially controversial topic. Turner and Antón^29^ suggested that giant hyenas were social, which allowed them to confront large predators and steal their prey. In contrast, the Venta Micena assemblage showed that the giant hyenas selectively transported certain parts of the carcass to their dens^42^. This behaviour supports the interpretation of solitary social behaviour because recent spotted hyenas transport all anatomical elements of the carcass to their den when scavenging in groups, but only selected parts when scavenging alone^42^. Moreover, the social behaviour of recent spotted hyenas is related to the expansion of the frontal region of the brain, a trait recently acquired in the *Crocuta* lineage^43^*.* Therefore, sociality may be a unique and recent acquisition in spotted hyenas.

Scavenging is a widespread behaviour among medium-sized carnivores in recent terrestrial ecosystems^40^, which is also practised by contemporary hunter-gatherers. Hadza obtained 20% of their meat through confrontational scavenging^17,23^. However, the consumption of carrion represents a “windfall” resource for Hadza foragers and not a regular activity due to some shortcomings, such as seasonal variations in encounter rates and the size and completeness of carcasses^44^. Wild chimpanzees also scavenge, but rarely. Anecdotal evidence of scavenging by chimpanzees has been reported from Gombe, Mahale, Taï, and Ngogo^21,45^. Confronting large carnivores is risky, but chimpanzees reduce this risk by increasing the number of participants and shouting and throwing stones to intimidate leopards. Scavenging, even passive scavenging, is risky. Indeed, the “fatal attraction” hypothesis^46^ proposes that carcass sites amplify the suppression effect of large carnivores on medium-sized carnivores. Despite being a widespread behaviour, scavenging has only been presented as a successful strategy for early hominins in the short term^33^. In contrast, the simulations show that scavenging could be an efficient and adaptive behaviour for the Epivillafranchian under certain conditions.

The results of the simulation experiments highlight the importance of group size for the viability of scavenging when competition is considered. Indeed, it can be argued that defending or stealing a carcass from other scavengers, as simulated in our experiments, does not differ from stealing a carcass from a predator. Interestingly, our results showed that when the group size of hominins was not sufficient to chase away their competitors, the hominins survived until the end of the simulation only when carcasses were abundant because of the high density of predators in a highly productive ecosystem (Fig. 1). This suggests that a fully passive scavenging strategy without direct confrontation with competitors would be energetically inefficient as a regular strategy (Fig. 2), although it could still be viable on an opportunistic basis. Hominins foraging alone or in very small groups could not rely on the active search for carrion as the main food resource, although they could feed on an abandoned carcass, which was found as a stroke of luck when foraging on other resources, until competitors appeared. In contrast, roaming around the landscape in search of carcasses would be an efficient behaviour for a group of hominins that was large enough to chase away other scavengers. Another important issue demonstrated in the experiments was the existence of an optimal group size for the foraging band (13 individuals in our simulations). The energetic cost of the scavenging activity increases with group size when the group is larger than the minimum size necessary to chase away all competitors and predators. This is because a group that is too large is not satiated by a single carcass and should expend energy in search of additional resources. Thus, the less productive the ecosystem and the scarcer the carcasses, the more energy-intensive this strategy is for a large group. However, it is important to note that the results of our simulations should not be interpreted as estimates of the viable population density of hominins or the optimum group size. The values obtained for these response variables are dependent on the values arbitrarily assigned to parameters such as the group size necessary to chase away giant hyenas and predators or the carrion waste rate. The results suggest the existence of an optimum group size but do not provide an estimate of it. In the real world, this optimum would be determined by the strength necessary to chase away competitors and by the size and nutrient content of the carcasses. The positive effect of foraging in a group with size close to the optimum is larger, and the encounter rate with competitors and predators is higher. Thus, foraging in a group of size close to the optimum is more beneficial in highly productive ecosystems, where the density of carnivores and the encounter rate are higher.

Moreover, scavenging large carcasses in competition with other carrion eaters may have led hominins to coordinate their movements, group cohesion, defence, cooperation, and communication. A relationship between scavenging and language emergence was proposed^47^. It has been suggested that cooperative behaviour also allowed rapid processing and disarticulation of large carcasses with stone tools to minimise the time spent at the kill site and reduce the encounter rate with carnivores^19,48^, but this behaviour was not included in our simulations. Direct competition between scavengers, in our case *Homo* and *Pachycrocuta brevirostris*, could favour grouping. A certain number of hominins banding together, even brandishing sticks or stones, and shouting could chase out larger predators from their preys^17,49^. Indeed, archaeological evidence from Fuente Nueva-3^50^ and Dmanisi^51^ suggests that cobbles and limestone blocks could be used as throwing stones to drive away predators and competitors, reducing the risk of the confrontation^52^. The results showed that maintaining an optimum group size can be an important factor for success in the competition for carrion in the form of interference competition^53^. Therefore, an optimum group size protects against predation ^45^ and improves scavenging efficiency.

SCAVCOMP-ABM^54^ simulated the trophic behaviour of hominins without using a central place-foraging model^55–58^. This is an important difference from other computer simulations of hominin foraging strategies, including simulations of scavenging activities^59,60^. In the HOMINIDS (Hungry Omnivores Moving, Interacting, and Nesting in Independent Decision-making Simulations) model^59^, hominin agents leave their nests in the morning and roam individually to search for food. If an agent finds an abandoned carcass, it feeds on it; however, if there are other scavengers on the spot, the hominin calls for help and waits until more hominins arrive to chase away competitors and feed on the spot. Szilágyi et al.^60^ developed an ELBA model and simulated confrontational scavenging to test a hypothesis regarding the emergence of language. Since carnivores are not included in the ELBA model^60^, it closely simulates passive scavenging rather than confrontational scavenging. In the ELBA model, hominins forage during the day and return to a campsite at night, where they share food and information regarding the location of the carcasses. Moreover, in the ELBA model, group size had no influence on the ability of hominins to access a carcass but did influence their capability to transport the carcass to the campsite. In contrast, in SCAVCOMP-ABM^54^, hominins live in small bands that move from one resource patch to another and remain in the patch until the resources (carrion) are depleted; this type of mobility is better described as an optimal patch-use strategy^61^. Similarly, a strategy without a central place or reference site and provisioning in a fission–fusion social model is common among non-human primates, such as chimpanzees and baboons^62–65^. Although a central place strategy is usually assumed for coeval hominin populations in Africa^56^, assuming a different behaviour for the European hominins during the Epivillafranchian does not conflict with the archaeological records. Most early Pleistocene sites from Iberia are interpreted as marginal occupations by hominins, as Fuente Nueva-3^32,39^ Barranco León D^2^, Vallparadís EVT7^66,67^, or Barranc de la Boella sites^68,69^, or as low intensity occupations as Sima del Elefante TE9^70^. Most of these sites are open-air localities usually associated with floodplains or riparian environments and are interpreted as foraging sites. Only the Atapuerca TD6.2 assemblage has been interpreted as a home base intensively occupied by hominins over long periods of time^71,72^ and might thus be evidence of a central place foraging strategy. However, TD6.2, which has been dated to approximately 0.8 Ma^73^, has a faunal assemblage characterised by the absence of both *P. brevirostris* and *Megantereon* and the presence of *Crocuta crocuta*^74^. Thus, Atapuerca TD6.2, which corresponds to the time after the extinction of *Megantereon* and the replacement of *Pachycrocuta brevirostris* by *C. crocuta*, is younger than that considered here and with an entirely different ecological scenario.

The quantitative estimates of carrion production support that sabre-toothed felids created a niche for scavengers by abandoning carcasses with a high nutrient content. In this scenario, scavenging was a reliable food procurement strategy for early hominins in southern Europe, as they foraged in groups strong enough to chase giant hyenas away from the carcasses. This suggests that the differentiation between passive scavenging and kleptoparasitism is limited in the presence of a strong competitor. However, group size had to be moderate in order to maximize the energetic efficiency of the activity. Scavenging does not require advanced technology only group cohesion and cooperation and was likely an important source of meat and fat for *Homo* sp. in Europe, especially in winter when plant resources were scarce.

## Methods

### SCAVCOMP-ABM

Several simulation experiments were performed using SCAVCOMP-ABM v.40.2^54^, an agent-based model (ABM) developed in Net Logo 6.2.2^75^ that could simulate the competition for carrion among hominins and carnivores in an early Pleistocene European ecosystem. An ABM is a computational modelling paradigm that simulates complex systems by encoding the behaviour of simulated entities (agents) in simple rules to observe the results of these agents’ interactions^36^. In this study, an overview of the SCAVCOMP-ABM is provided; however, a full description of the model following the ODD (Overview, Design concepts, and Details) protocol^76^ is provided elsewhere^54^. The terminology of Montgomery^77^ was followed in this study, where the parameters that may be changed between simulation experiments are called “factors”, the factor values are called “levels”, parameters that are fixed are called “constants”, and the output variables are called “responses”. Therefore, each experiment was defined by a combination of the initial levels of the factors. By comparing the results of the different experiments, the effects of these factors on the response variables were observed.

SCAVCOMP-ABM was designed to evaluate the viability of scavenging as a foraging strategy for hominins under different carnivore guild compositions and different group sizes. The environment of the model consisted of a grid of 51 × 51 cells (patches) representing an area of 2601 km^2^ in a homogeneous landscape. Each patch had an area of 1 km^2^. The agents of the model were groups of hominins and packs or individuals belonging to the large carnivore species present in the late Villafranchian and Epivillafranchian^8,78^. These agents were classified as predators (*Homotherium latidens*, *Meganthereon* sp., and *Panthera gombaszoegensis*) or scavengers (*Homo* sp. and *Pachycrocuta brevirostris*). SCAVCOMP-ABM allows the inclusion of a third type of agent called a hybrid, which shares characteristics with both predators and scavengers; however, this type was excluded in these simulations. The composition of the carnivore guild changed among experiments by excluding some of the species listed above and modifying the initial densities of the species included. The agents in each group were defined by a separate set of state variables, and each species was defined by a distinct set of values for the state variables. The behaviour of the agents was determined by a set of rules, which were different for predators and scavengers. All agents of the same species were identical; that is, they were defined by the same values for all their state variables.

This model simulated the dynamics of a community over a year or a few years. Each tick represents an hour and agents did not reproduce. Predators moved randomly around the environment and stochastically produced carcasses. The frequency of production and the amount of nutrients in the carcass (energy) differed for each predator species, and hunting was not simulated using this model. The energy contained in the carcass decreased with time at a rate determined by the carrion-wastage-rate (kcal/day). The carrion-wastage-rate simulated the “natural decay” of the energetic content of a carcass abandoned in the landscape. In the real world, decay occurs through the action of microorganisms, and because carcasses were consumed by other species (birds, invertebrates, or other carnivores), this was not simulated. Scavengers aimed to collect carrion and obtain energy to compensate for their energy losses. Scavengers spent energy continuously at a basal rate and at a higher rate when moving. The energy expenditure rate differed for each species. If the energy of the scavenger agent declines to zero, the agent dies. Thus, the objective of scavengers was to balance energy expenditure and gain energy for survival. Survival success was measured based on the total population of the scavenger species at the end of the simulation.

Direct competition, or confrontation, is simulated in the model by allowing only one agent to stay in a patch. If two or more agents coincide in the same patch, only one of the agents with the highest *rank* stays in the patch and the rest of agents move away. The *rank* is an attribute of the species. All the agents of the same species have the same *rank*. All predators have a *rank* value of 5, but the *rank* of the scavengers depends on the size of the pack as follows:

$$Pachycrocuta\;brevirostris\;{\text{rank }} = {\text{ pack}} - {\text{size}} \times {2}.{5}$$

$${\text{Hominins}}\;{\text{rank = pack - size/2}}$$

An agent representing a solitary *P. brevirostris* had a lower rank than any predator but a higher rank than a group of hominins with less than five individuals. Groups of six or more hominins had a rank higher than any other agent. These values were selected arbitrarily; however, they reflected the effect of group size on confrontations between hominins and carnivores. Ethnographical observations indicate that less than five adult Hadza are able to chase away any large felid^23^, and a group of ten chimpanzees has been reported to confront a leopard at Mahale (Tanzania)^45^. Direct competition for a single carcass between two agents was simulated by allowing only one of them to stay in the patch containing carrion, even if they were agents of the same species, for example, two different groups of hominins. Moreover, it was assumed that scavengers avoided direct interactions with predators unless they could chase them away (because the predator had a lower rank). However, notably, this model did not intend to simulate kleptoparasitism or confrontational scavenging^17–19,79^.

### Parameterization

The default levels of the SCAVCOMP-ABM parameters are listed in Supplementary Table S2. The levels of many parameters were derived based on theoretical or real-world observations. However, several parameters were assigned values after observing the effect of their variation on the behaviour and results of the model. The levels of initial-nutrition-state*,* range-of-view, and daily-carrion-wastage-rate could not be determined from theory or derived from the real world, and therefore were based on the sensitivity analysis results, as shown in Supplementary Methods S3.

#### Predators and carrion availability

The number of energetic resources available in the environment in the form of scavengeable carcasses depends on the frequency of carcass production by predators, the average size of the carcasses produced by each predator, the population density of the predator^23^_,_ and their food intake rate. Carcass size was determined based on the size of the prey preferred by each predator (Table 3). A discussion on the feeding preferences of large Epivillafranchian carnivores was provided by Rodríguez et al.^26^. In this study, the body weight of the preferred prey was obtained as the average body weight of the two prey species representing more than 80% of the predator’s diet, as indicated by isotopic analyses of the fossils of these species^80^.

**Table 3:** Estimates of population densities of large carnivores in Iberian localities during the end of the Late Villafranchian and Epivillafranchian, and initial values used in the simulation experiments.

	*H. latidens*	*M. whitei*	*P. gombaszoegensis*	*P. brevirostris*
Estimated population density (individuals/100 km^2^)				
Iberia^8^	3.5 (2.0–6.1)	8.7 (5.0–15.0)	5.4 (3.1–9.4)	7.1 (4.1–12.3)
Fuentenueva-3 & Barranco León D^86^	3–4	4–5	–	4–6
Venta Micena^85^	3–5	5–6	5–6	6–7
Population density (individuals/100 km^2^) in the simulation				
Low predator density Experiment	2	5	3	12
Average predator density Experiment	3.5	8.7	5.4	12
High predator density Experiment	6	15	9	12

The estimations of carcass production rates were based on recent reports of the feeding behaviour of large carnivores (Supplementary Methods S2). In the simulations, the hunting frequencies were assumed to be 1 kill per 8 days for *Homotherium* and 1 kill per 7 days for *Megantereon* sp. and *Panthera gombaszoegensis*. The equation provided by Farlow^81^ was used to estimate the daily energy requirements of the predators based on their estimated average body masses^13^. The amount of energy consumed by the predator before abandoning the carcass was obtained by multiplying the daily energy requirements by the average interval between kills (in days). A kill should fulfil the energetic requirements of the predator until the next kill. Thus, assuming low killing ratios, we obtained conservative estimates of carcass production rates and carcass energy content for these predators. The edible mass of a complete carcass was obtained by multiplying the average prey body weight by the wastage factor given by Viljoen^82^. This wastage factor is dependent on body size, as follows: < 50 kg, 80% edible; 50–150 kg, 75% edible; 151–250 kg, 70% edible; 251–500 kg, 65% edible; 500–1000 kg, 60% edible; > 1000 kg, 55% edible. The amount of energy that could be obtained from an entire carcass was then computed by multiplying the edible mass by a conversion factor of 1300 kcal/kg. This conversion factor was based on the caloric content of a kilogramme of the edible mass of different ungulates^83,84^. Finally, the energy content of the carcass produced by a predator was obtained by subtracting the energy consumed by the predator from the edible mass of the entire carcass.

The published estimates of sustainable population densities of large carnivores in Mediterranean ecosystems during the late Early Pleistocene are reviewed in Table 3^85,86^. All these estimations are roughly coincident, although they are based on different methodologies. In our simulations, the estimates of the lowest, maximum, and average population densities^8^ were used to represent the three different conditions. These conditions can be interpreted as three different ecosystems with a gradient of increasing primary production, with the most productive ecosystems sustaining higher population densities.

#### Scavengers and energy requirements

The energy requirements of any mammal are largely determined by its body size^87^. Therefore, the well-known relationship between body size and metabolic rate was used in this simulation to estimate the basal metabolic rates of humans^88^ and giant hyenas^89^; the details of which are provided in Table 4. The body weight of *Pachycrocuta brevirostris* was obtained from Rodríguez-Gómez et al.^85^. The body weight of the hominins was assumed to be 62 kg, which is the estimated body weight of *Homo ergaster* and *Homo erectus*^90^. The daily energy requirements of hominins (DER), which were used in the model to estimate the energetic cost of movement, were set to 3000 kcal/day. This energy expenditure was similar to the estimated cost of walking for *H. ergaster*^91^. Locomotion is energetically costly for giant hyenas because of their shortened distal limbs^29^. Thus, the DER of the giant hyena was set at three times the estimated BMR (basal metabolic rate).

**Table 4:** Values of the energetic parameters of hominins and giant hyenas. Daily energy requirements (DER) of the giant hyena was set at three times the estimated basal metabolic rate (BMR).

	Hominin	*P. brevirostris*
Body weight (kg)^85,90^	62	110
BMR (kcal/day)^88,89^	1547	1734
DER (kcal/day)	3000	6175

In the simulations, the giant hyenas were assumed to be solitary scavengers^10,30,92^. Therefore, the pack-size was set to one. However, it has also been proposed that the giant hyena would require group action to defend the carcasses from other predators and practise confrontational scavenging^29^. According to this alternative interpretation, social behaviour would allow giant hyenas to complete their diet by hunting their own prey; however, this was not addressed in this study.

The velocity parameter determines the speed at which agents move when searching for food or avoiding an enemy. In the simulation, hominins moved at 5 km/h, corresponding to the medium walking speed of humans currently^93^, and the same velocity was used for giant hyenas because of the lack of an estimate of the speed at which they moved, as it is generally accepted that they were not highly cursorial carnivores^29,30^. Notably, the velocities represented in the model were the average speeds when moving long distances and not at a fast running speed.

The initial population densities of hominins (14 individuals/100 km^2^) and giant hyenas (12 individuals/100 km^2^) were based on the values observed for living species and published estimates. The ecological density of hominins in Barranco León D and Fuente Nueva-3 was estimated to be in the range of 10–14 individuals/100 km^2^ if they follow a pure scavenging strategy, 9–10 individuals/100 km^2^ if they are hunters, and 10–12 individuals if they adopt a hunting-scavenging strategy^86^. The densities of recent hunter-gatherer groups from mid-latitude areas (30–50°N) range from 0.4 to 300 individuals/100 km^2^^94^. Estimated sustainable densities of 4.1–12.2 individuals/100 km^2^ were estimated for *P. brevirostris* in the Iberian Peninsula during the late Early Pleistocene^8^. Estimated sustainable densities for the giant hyena were 4–6 individuals/100 km^2^ at Barranco León D and Fuente Nueva-3^86^, and 6–7 individuals/100 km^2^ at Venta Micena^85^. The initial densities were set to the upper limit of the estimated intervals because the simulations proceeded by reducing the number of groups or packs in the scavenger guild until a stable configuration was reached or, alternatively, until the system collapsed.

### Experiment design

In this study, the competition between *P. brevirostris* and hominins in the late Early Pleistocene of the Iberian Peninsula and the role of sabre-tooths as carrion producers were focused. The large carnivorous guilds of Iberia during the Epivillafranchian included *Canis etruscus, Lycaon lycaonoides, Homotherium latidens, Megantereon whitei, Panthera gombaszoegensis,* and *Pachycrocuta brevirostris*^8^. Archaeological records show that the first European hominins coexisted in the same community as two sabre-toothed felids (*H. latidens* and *M. whitei*), Pleistocene wild dogs (*Lycaon lycaonoides*), small canid (*Canis etruscus/mosbachensis*), and giant hyena in localities such as Fuente Nueva-3 and Barranco León D^86^. The European jaguar (*P. gombaszoegensis*) was recorded together with the two sabre-toothed felids and the giant hyena in several palaeontological assemblages of the late Villafranchian or Epivillafranchian age, including Ceyssaguet-1^95^, Monte Argentario^96^, Olivola^97^, Untermassfeld^98^, Venta Micena^85^, and Cueva Victoria^99^. Therefore, the experiments were conducted under two different ecological scenarios. In Scenario 1, the predator guild was composed of *H. latidens, M. whitei,* and *P. gombaszoegensis,* whereas in Scenario 2, only two sabre-toothed felids comprised the guild. Under both scenarios, the scavenger guild comprised *Homo sp.* and *P. brevirostris*.

Furthermore, *Canis etruscus* was excluded from our simulation experiments because this small canid is unlikely to be a large carcass producer. *Lycaon lycaonoides* was also excluded as a significant carcass producer because the early Pleistocene wild dogs were assumed to possess a behaviour similar to that of the recent African wild dogs (*L. pictus*)*.* A pack of African wild dogs can effectively eat an entire carcass in a short time, unless disturbed by hyenas or other carnivores attempting to steal their kill^100^. Thus, it was assumed that *L. lycaonoides* did not abandon carcasses containing a significant amount of nutrients and were not included in the experiments. Moreover, *C. etruscus* probably consumed carrion opportunistically but was also excluded from the experiments as an agent for the sake of simplicity. Instead, the effect of its scavenging activity on the availability of carcasses was summarised in the carrion-wastage-rate parameter.

The experiments evaluated the effects of two factors (size of the hominin group and population densities of predators and scavengers) on the performance of hominins as passive scavengers in two ecological scenarios. The two tested factors were ultimately dependent on the primary production of the ecosystem^8^. The effect of these two factors was evaluated on two response variables: the total population of hominins and hyenas at the end of the simulation experiment and the average daily energy expenditure by a single hominin and *P. brevirostris*. The daily energy expenditure is a measure of the energy cost of a scavenging strategy. It was assumed in the framework of the Optimal Foraging Theory^101,102^ that the best feeding strategy is that which allows an individual to survive at the lowest cost, and allocate more energy to other activities such as reproduction.

We performed simulation experiments with hominin group sizes of 1, 4, 7, 10, 13, 16, 19, 22, and 25 individuals at low, medium, and high densities of predators (see the Parameterization section). This yielded 27 simulations for each scenario. All simulations were performed for 9000 ticks, representing 9000 h or 375 days. Since the SCAVCOMP-ABM includes a certain degree of stochasticity^54^, a previous analysis was necessary to determine the number of runs required to obtain a valid estimate of the response variables. A replication assessment was conducted prior to the experiments^103^ performing 120 runs of the model with default parameter levels (Supplementary Methods S1). After the results of the replication assessment were obtained, all simulations were replicated 70 times.

### Supplementary Information

Supplementary Information 1.

Supplementary Information 2.

## Data Availability

All data generated or analysed during this study are included in this published article [and its supplementary information files]. The output of simulation experiments is available as Supplementary Dataset 1. The SCAVCOMP-ABM v40.2 and ODD Protocol are available at 10.6084/m9.figshare.22716427.
