# Supplementary material for: Computer simulation of scavenging by hominins and giant hyenas in the late Early Pleistocene
Source: Sci Rep. 2023 Sep 28;13:14283. doi: 10.1038/s41598-023-39776-1 (PMC10539305; doi:10.1038/s41598-023-39776-1)
Supplement: Supplementary file 2 — Supplementary Information 2. [file 41598_2023_39776_MOESM2_ESM.pdf]

Supplementary Information for

Computer simulation of scavenging by hominins and giant hyenas in the late Early Pleistocene

Jesús Rodríguez[1]

Ericson Hölzchen[2,3]

Ana Isabel Caso-Alonso[4]

Jan Ole Berndt[3]

Christine Hertler[5, 6]

Ingo J. Timm[2,3]

Ana Mateos[1]*

[1] National Research Center on Human Evolution (CENIEH), Paseo Sierra de Atapuerca 3, 09002 Burgos, Spain.

[2] Chair for Business Informatics 1, Trier University, Behringstraße 21, 54296 Trier, Germany.

[3] German Research Center for Artificial Intelligence (DFKI). Smart Data and Knowledge Services - Cognitive Social Simulation. Trier University, Behringstraße 21, 54296 Trier, Germany.

[4] Facultad de Ciencias. Edificio de Biología. Universidad Autónoma de Madrid. c/ Darwin, 2. Campus de Cantoblanco. 28049 Madrid, Spain.

[5] The Role of Culture in Early Expansion of Humans (ROCEEH), Senckenberg Research Institute, Senckenberganlage 25, 60325 Frankfurt am Main, Germany.

[6] The Role of Culture in Early Expansion of Humans (ROCEEH), Heidelberg Academy of Sciences, Karlstraße 4, 69117 Heidelberg, Germany.

*Corresponding Author

Ana Mateos (ana.mateos@cenieh.es)

Content

Supplementary Note S1. Ecological background of scavenging in the Epivillafranchian 3

Supplementary Methods S1. Replication Assessment 5

Supplementary Methods S2. Carcass production by large predators 8

Supplementary Table S2. Default levels of the parameters 9

Supplementary Methods S3. Sensitivity analysis 10

References 15

Supplementary Note S1

Ecological background of scavenging in the Epivillafranchian

The Mediterranean ecosystems of the Epivillafranchian would grant hominins access to a variety of animal and plant food resources [1,2]. The trophic resources potentially available for the first European hominins include the flesh and bone grease from carcasses left behind by predators [3], hunting ungulates [4] and small animals [5], and gathering the abundant edible plants [1]. Thus, the food procurement strategies of these early hominins were likely wide ranging.

Nevertheless, the relationship between the structure of the carnivore guild and the survival opportunities for hominins is complex and involves positive and negative interactions. Factors as primary production, carrying capacity, and diversity of carnivores would have influenced the efficiency of the hunting and scavenging capabilities of hominins in the European Early Pleistocene. The late Early Pleistocene ecosystems of Southern Europe were structured with a moderate herbivore biomass, and a diverse, but not abundant, carnivore guild [6]. Carnivore species richness was high, but carnivores occurred at low population densities due to the relatively low carrying capacity of the Mediterranean ecosystems in comparison with recent tropical or subtropical ecosystems [2,6,7]. The relatively low ungulate biomass suggests that intraguild competition for trophic resources among secondary consumers was high in the ecosystems of the late Villafranchian (2.0-1.2 Ma) and the Epivillafranchian (1.2-0.8 Ma) from Southern Europe [6]. In contrast, the relatively low population densities of carnivores imply a low encounter probability of carnivores and hominins, reducing the risk of predation or direct confrontation. Nevertheless, the relationship between hominins and carnivores was likely not only competitive, but also commensal [8]. Different scenarios would set on the opportunities of procurement of animal resources (fat, bone grease and lean meat) for hominins. Furthermore, the relatively low ungulate biomass of the Mediterranean ecosystems [6] would affect scavengers and predators equally.

The diverse Epivillafranchian carnivore guild, including a variety of predators and scavengers adapted to a diversity of environments [9,10], affected the scavenging opportunities for hominins in a complex way. The sabre-toothed cats are represented in the Epivillafranchian by the genera *Megantereon* and *Homotherium* [2]. *Megantereon* is generally considered as a solitary ambush hunter in forest environments, especially riparian forests [11]. Moreover, the isotopic analyses suggest that *Megantereon* hunted mixed-feeding cervids in closed habitats [12]. The fossil record of Venta Micena shows that its main prey were *Soergelia minor*, *Equus altidens*, and *Praemegaceros verticornis* [13] and their preferred prey were in the range from 90 to 360 kg [2]. In contrast, the behaviour of *Homoterium latidens* is more controversial, especially concerning its social behaviour. Anatomical and morphofunctional analyses suggest that *Homoterium* was a hunter of large prey [14,15]. Unlike pantherines, *Homotherium* was well adapted to long-distance travel in open environments by its particular postcranial skeleton [11]. Isotopic analyses of *Homotherium* fossils from Venta Micena suggest that juvenile *Mammuthus Bison sp.*, and *Equus altidens* [13] were part of its diet, as a confirmation that this sabre-toothed cat was specialized in killing large prey. Sociality has been proposed for *Homotherium* [11] on the basis of the minor capability of a single individual to subdue and retain large prey; and as a requisite to defend their prey from giant hyenas. However, sociality is an extremely rare behaviour in living felids, restricted to the African lion, [16] and strong evidence for a social behaviour in *Homotherium* is lacking at this time. These two sabre-toothed cats likely played an important role in the

top-down regulation of Pleistocene ecosystems, and it has been proposed that they were essential to create a niche for scavengers in Europe.

The archaeo-palaeontological evidence from 'Ubeidiya [17,18] and Barranco León[19,20] seems to support scavenging as the main procurement strategy for the first human settlers of Eurasia. However, the evidence of primary access to carcasses at Sima del Elefante-TE9c has been interpreted as indicative of active hunting [4]; although early access to carcasses does not necessarily imply hunting. Therefore, Early Pleistocene European hominins were likely facultative scavengers at variable rates, able to subsist on other food resources in the absence of carrion. Moreover, they did not show competitive exclusion with other members of the carnivore guild, indicating that their niche did not completely overlap that of any other species [21].

As scavengers, hominins would have benefited from a relatively low encounter rate with predators in Southern Europe, while taking advantage of the abundant edible resources found in the carcasses abandoned by carnivores, due to satiation or to their morphofunctional limitations to extract within-bone nutrients [22]. Scavenging the kills of sabre-toothed cats (*Megantereon*, and *Homoterium*) was feasible for hominins because felids deflesh the carcasses of their prey, but have less ability to access within-bone nutrients than hyenids and canids, which consume carcasses more thoroughly [23-25] (but see also Domínguez-Rodrigo, et al.[26]). Moreover, the nutrient content of the carcasses abandoned by sabre-tooths would be especially high, and include also flesh, if they consumed the carcasses only partially. Confrontational scavenging, or kleptoparasitism, is common in recent spotted hyenas, which are able to chase away leopards, cheetahs, hunting dogs, and even lions if the pack of hyenas is large enough, to steal their kills [27]. A similar behaviour has been proposed for the giant hyena [28] and for early hominins [29]. Undoubtedly, this type of behaviour entails a certain type of cooperation. A relatively large group size would have been necessary to deal with predators in order to get a successful result on scavenging [29]. Moreover, it has been suggested that confrontational scavenging could have driven the evolution of language and social cooperation [30].

Supplementary Methods S1

Replication Assessment

SCAVCOMP-ABM includes a certain degree of stochasticity. Thus, a precision analysis was performed in order to determine the number of runs required to obtain a valid estimation of the response variables. Precision analysis of SCAVCOMP-ABM version 40.2 was carried out by running 120 simulations with the default values of the parameters (Supplementary Table S1) during 9,000 ticks (representing 9,000 hours or 375 days).

Supplementary Table S1. Default values of the parameters.

Parameter	Default
initial-available-carrion-units	10,000
hours-without-scavengers	50
initial-carrion-probability	0
daily-carrion-wastage-rate	100,000
range-of-view-all	3
initial-nutrition-state	"middle condition"
stop-at-hours	true
stop-at-hours-value	9,000
stop-at-no-more-scavengers	true
velocity-p_brevirostris	5
der-p_brevirostris	6,174.7
bmr-p_brevirostris	1,734
initial-density-p_brevirostris-100km^2	6
pack-size-p_brevirostris	1
initial-density-hominins-100km^2	5
der-hominins	3,000
pack-size-hominins	5
velocity-hominins	5
bmr-hominins	1,547
initial-density-homotherium-100km^2	3
homotherium-carrion-units-production-rate-per-hour	0.0052
carrion-units-from-prey-by-homotherium	107,000

Parameter	Default
pack-size-homotherium	1
pack-size-panthera	1
initial-density-panthera-100km^2	5
panthera-carrion-units-production-rate-per-hour	0.006
carrion-units-from-prey-by-panthera	36,156
carrion-units-from-prey-by-lycaon	0
initial-density-lycaon-100km^2	0
pack-size-lycaon	10
lycaon-carrion-units-production-rate-per-hour	0.041
meganthereon-carrion-units-production-rate-per-hour	0.006
initial-density-meganthereon-100km^2	8
carrion-units-from-prey-by-meganthereon	118,800
pack-size-meganthereon	1
bmr-canis_etruscus	330.4
carrion-units-from-prey-by-canis_etruscus	5,200
canis_etruscus-carrion-units-production-rate-per-hour	0.000
initial-density-canis_etruscus-100km^2	0
pack-size-canis_etruscus	1
velocity-canis_etruscus	1
der-from-carrion-canis_etruscus	0.2
der-canis_etruscus	1,241
der-from-carrion-crocuta_crocuta	0.2
carrion-units-from-prey-by-crocuta_crocuta	421,669.138
initial-density-crocuta_crocuta-100km^2	0
velocity-crocuta_crocuta	1
der-crocuta_crocuta	1,349.13
crocuta_crocuta-carrion-units-production-rate-per-hour	0.0058
pack-size-crocuta_crocuta	3
bmr-crocuta_crocuta	1,120.8

Six response variables were considered:

1. Final number of hominin packs
2. Final number of *P.brevirostris* packs
3. Average daily energy spent by hominin pack
4. Average daily energy spent by *P. brevirostris* pack
5. Average daily energy gained by hominin pack
6. Average daily energy gained by *P. brevirostris* pack

Precision after the *n* simulation is based on the confidence interval and is computed as [31]:

$$d_n = \frac{t_{n-1,\alpha/2} \cdot \frac{S_n}{\sqrt{n}}}{\bar{X}_n}$$

Where *n* is the number of simulation runs $t_{n-1,\alpha/2}$ is the Student's t distribution quartile, S_n is the cumulative standard deviation, and $\bar{X}_n$ is the cumulative mean. The critical value was established as 0.05 to balance the statistical validity and the simulation performance [32].

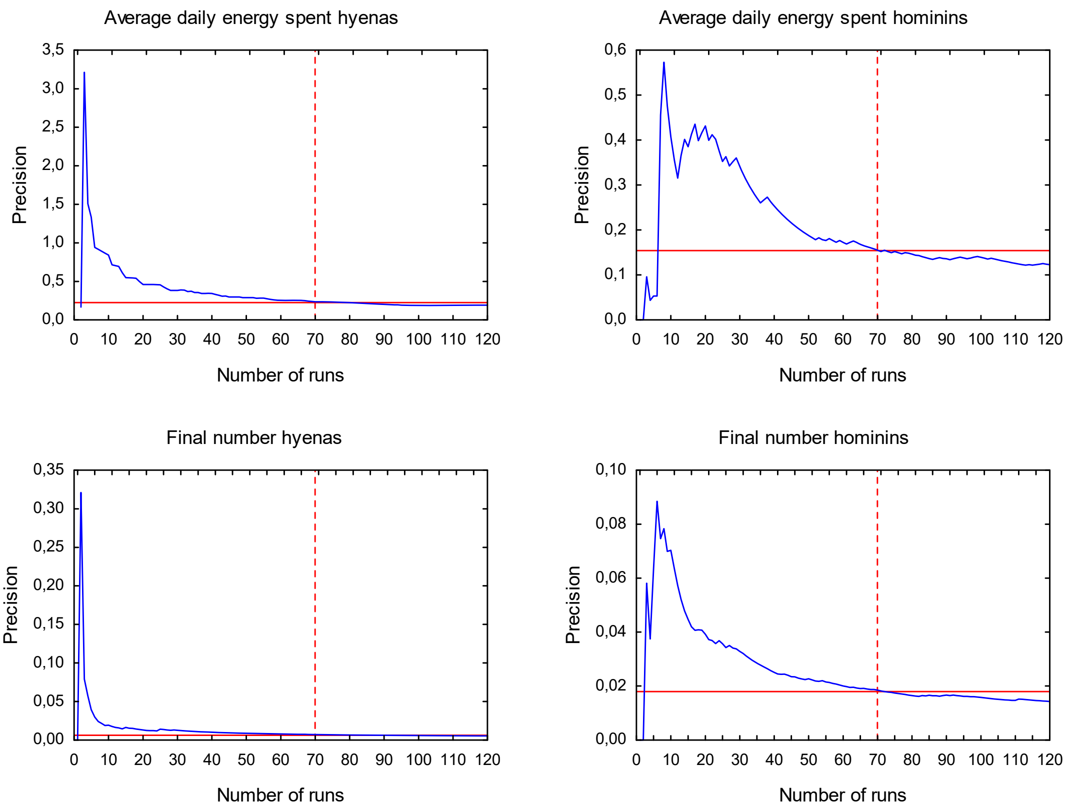

Supplementary Figure S1. Results of the replication assessment after 120 runs. The vertical dashed line marks the selected number of runs and the horizontal line the accepted precision.

Supplementary Methods S2

Carcass production by large predators

Carcass production rate was determined on the basis of the behaviour of recent large predators. Big cats typically make a large kill approximately every week, eat for a few days and then go several days without eating. According to Sunquist and Sunquist [33], females with kittens hunt at a higher frequency (1 kill every 8-9 days in tigress, and 1 kill every 10 days in cougars) than singleton females (1 kill every 5-6 days in tigress, and 1 kill every 6.8 days in cougars). Leopards kill a large prey every 5-7 days [33]. Big cats consume a huge amount of meat at a single sitting (20% of their own weight in a single night). It has been reported that a tigress spent 3 days with its kill and consumed an average of 15 to kg per night while leopards consumed 3-5 kg of meat per day and cheetahs consumed 2-4 kg/day [33]. Laundré [34] estimated the daily requirements of cougars as 3,143.7 kcal for males, 2,705.4 kcal and 2,420.0 kcal for females and Smith, et al. [35] point out that a cougar needs to kill a single prey the size of a mule deer (*Odocoielus virginianus*) or a vicuña (*Vicugna vicugna*) every two weeks to satisfy its energetic requirements. However, recorded killing rate is usually twice that figure [35]. This is explained because cougars eat the carcass only partially, before abandoning it while still containing a significant amount of nutrients. Nevertheless, field observations [36] show that the amount of edible resources led in a carcass by carnivores depends on the size of the prey and the identity of the predator. Scavenging a wildebeest or a zebra killed by lions may yield up to 5.5 kg and 15.2 kg of meat respectively, taking apart the bone marrow content [36]. Carcasses of ungulates between 114 kg and 900 kg killed by lions still present significant amounts of meat, although smaller animals are consumed almost completely. Pack size is a key factor determining the amount of resources led on the carcasses abandoned by social carnivores. It has been shown that the number of wolves in a feeding pack is negatively correlated to the amount of resources led in the carcass [37]. Packs of African wild dogs (*Lycaon pictus*) consume their kills promptly, and abandon carcasses containing almost no edible portions [38,39].

It has been estimated that large carnivores provide on average 1,351 kg of carrion per individual per year to scavengers [40] in recent ecosystems. This means that a single large predator produces 25.9 kg of carrion per week or, translated into energy units with an equivalence of 1,300 kcal/kg[41], 33,670 kcal/week.

Supplementary Table S2

Supplementary Table S2. Default levels of the parameters of SCAVCOMP-ABM.

Agent	Parameter	Default
	initial-available-carrion-units	10,000 kcal
	hours-without-scavengers	50 h
	initial-carrion-probability	0
	daily-carrion-wastage-rate	100,000 kcal/day
	stop-at-hours	true
	stop-at-hours-value	9,000 h
	stop-at-no-more-scavengers	true
All	range-of-view-all	3 km
All	initial-nutrition-state	"middle condition"
giant hyena	velocity-p_brevirostris	5 km/h
giant hyena	der-p_brevirostris	6,174.7 kcal/day
giant hyena	bmr-p_brevirostris	1,734 kcal/day
giant hyena	initial-density-p_brevirostris-100km^2	6 ind/100km^2
giant hyena	pack-size-p_brevirostris	1
hominin	initial-density-hominins-100km^2	5 ind/100km^2
hominin	der-hominins	3,000 kcal/day
hominin	pack-size-hominins	5
hominin	velocity-hominins	5 km/h
hominin	bmr-hominins	1,547 kcal/day
Homotherium	initial-density-homotherium-100km^2	3 ind/100km^2
Homotherium	homotherium-carrion-units-production-rate-per-hour	0.0052 carcass/h
Homotherium	carrion-units-from-prey-by-homotherium	107,000 kcal/carcass
Homotherium	pack-size-homotherium	1
jaguar	pack-size-panthera	1
jaguar	initial-density-panthera-100km^2	5 ind/100km^2
jaguar	panthera-carrion-units-production-rate-per-hour	0.006 carcass/h
jaguar	carrion-units-from-prey-by-panthera	36,156 kcal/carcass
megantereon	meganthereon-carrion-units-production-rate-per-hour	0.006 carcass/h
megantereon	initial-density-meganthereon-100km^2	8 ind/100km^2
megantereon	carrion-units-from-prey-by-meganthereon	118,800 kcal/carcass
megantereon	pack-size-meganthereon	1

Supplementary Methods S3

Sensitivity analysis

Although the values of several parameters of the model were derived from real world data, a number of parameters had to be assigned values after observing the effect of their variation on the behaviour and results of the model. The effect of different levels of initial-nutrition-state, range-of-view and daily-carrion-wastage-rate on the final number of hominins and hyenas and on the energetic expenditure of both of them were tested. The analyses were performed with 70 runs of the model for each level of the parameter to be tested, or for each combination of parameters.

The five levels of Initial-nutrition-state ("bmr condition", "bad condition", "middle condition", "good condition", "random condition") were tested by running the model 350 times (5 levels x 70 replications). The values "bmr condition" and "bad condition" produce a final number of hominin packs lower than the other levels of the parameter (Supplementary Figure S2A). The final number of giant hyena packs is only affected by "bmr condition". This effect is entirely due to deaths occurred during the first ticks. The energy variables are not affected by changes in the initial value of the parameter (Supplementary Figure S2B). Thus, we selected "middle condition" as the default level for our simulation experiments.

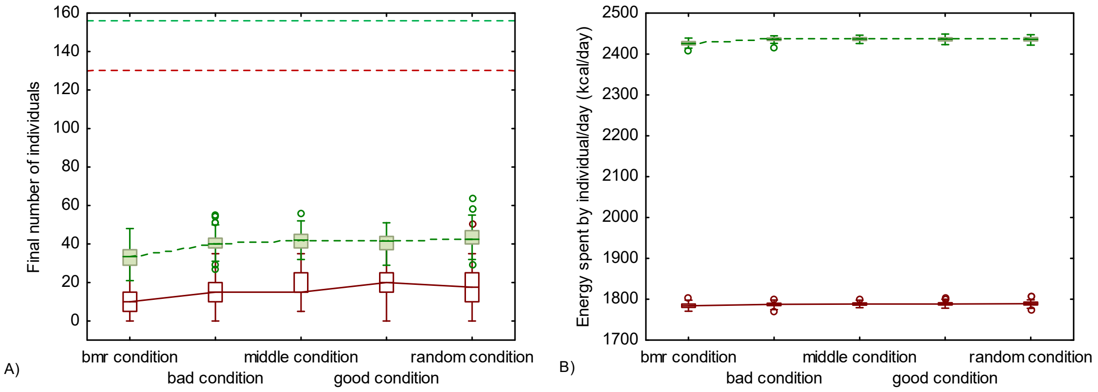

Supplementary Figure S2. Effect of the initial-nutrition-state on the final number of hyenas and hominins (A), and energetic expenditure of both species (B) shown by 70 runs of the model for each level. The initial number of hominins (red) and hyenas (green) are indicated by horizontal dashed lines. The limits of the boxes correspond to the first and third quartiles; the median is shown with a horizontal line. The whiskers mark the maximum and minimum without outliers and extreme values. Outliers are indicated with a white dot.

We analysed two factors by combining 6 levels for range-of-view (1, 2, 3, 4, 5, and 6 km) with 8 levels for daily-carrion-wastage-rate (25,000; 50,000; 75,000; 100,000; 125,000; 150,000; 175,000 and 200,000 kcal/day). This gives 3,360 replications of the model (6 levels x 8 levels x 70 replications).

Low values of range-of-view (< 3) made scavengers prone to extinction, especially when carrion-wastage rate was > 50,000 kcal/day (Figure S3). When the range-of-view was equal to or higher than 3 km, energetic expenditure was negatively correlated with range-

of-view in hominins (Spearman Rho = -0.14, p<0.001) but positively correlated in *P. brevirostris* (Rho = 0.37, p<0.001; Figure S4).

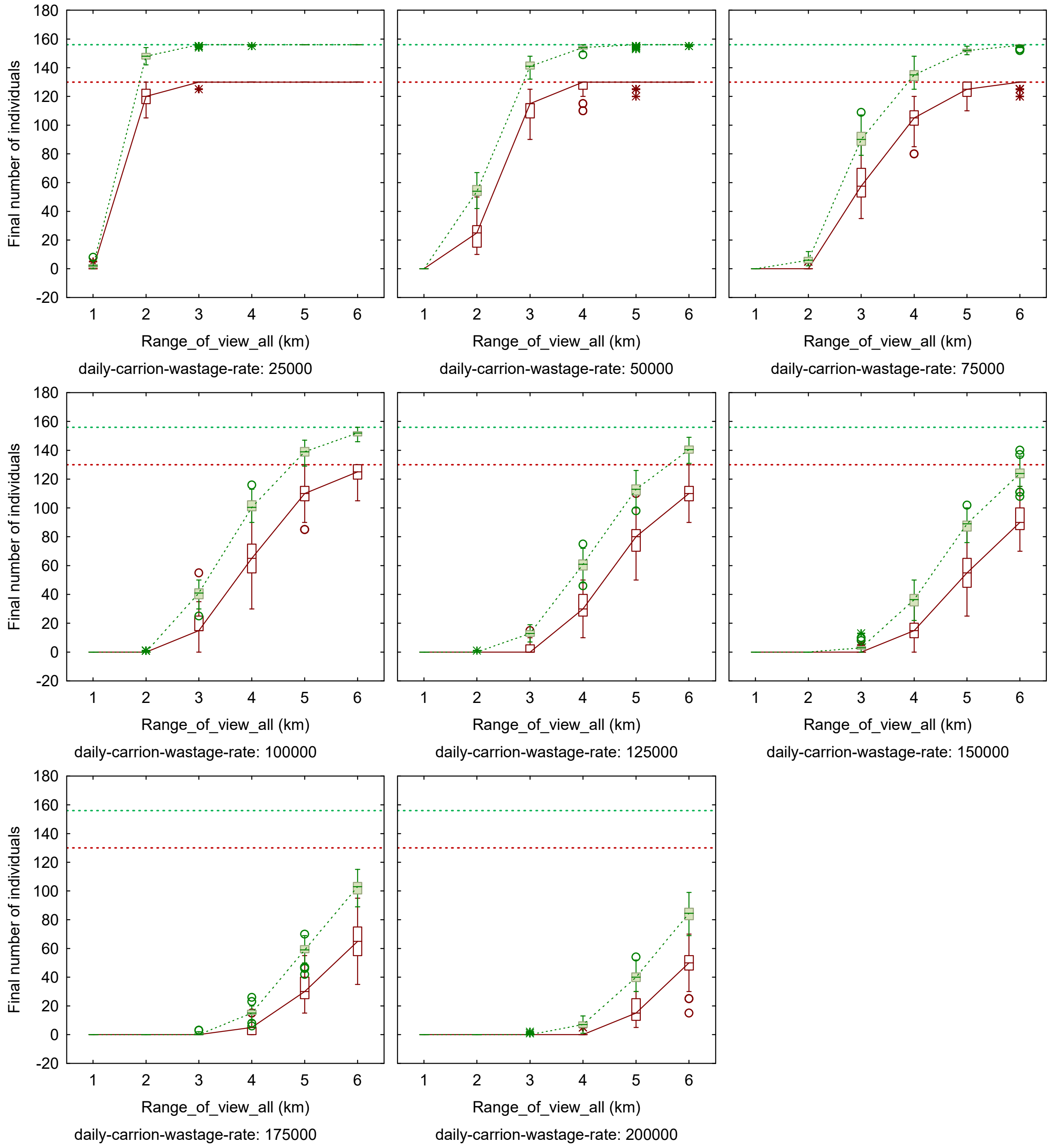

Supplementary Figure S3. Effect of range-of-view on the final number of hominins (red line) and hyenas (green line) at different levels of daily wastage rate. The initial number of hominins (red) and hyenas (green) are indicated by horizontal dashed lines. The limits of the boxes correspond to the first and third quartiles; the median is shown with a horizontal line. The whiskers mark the maximum and minimum without outliers and extreme values. Outliers and extreme values are indicated with a white dot and an asterisk, respectively.

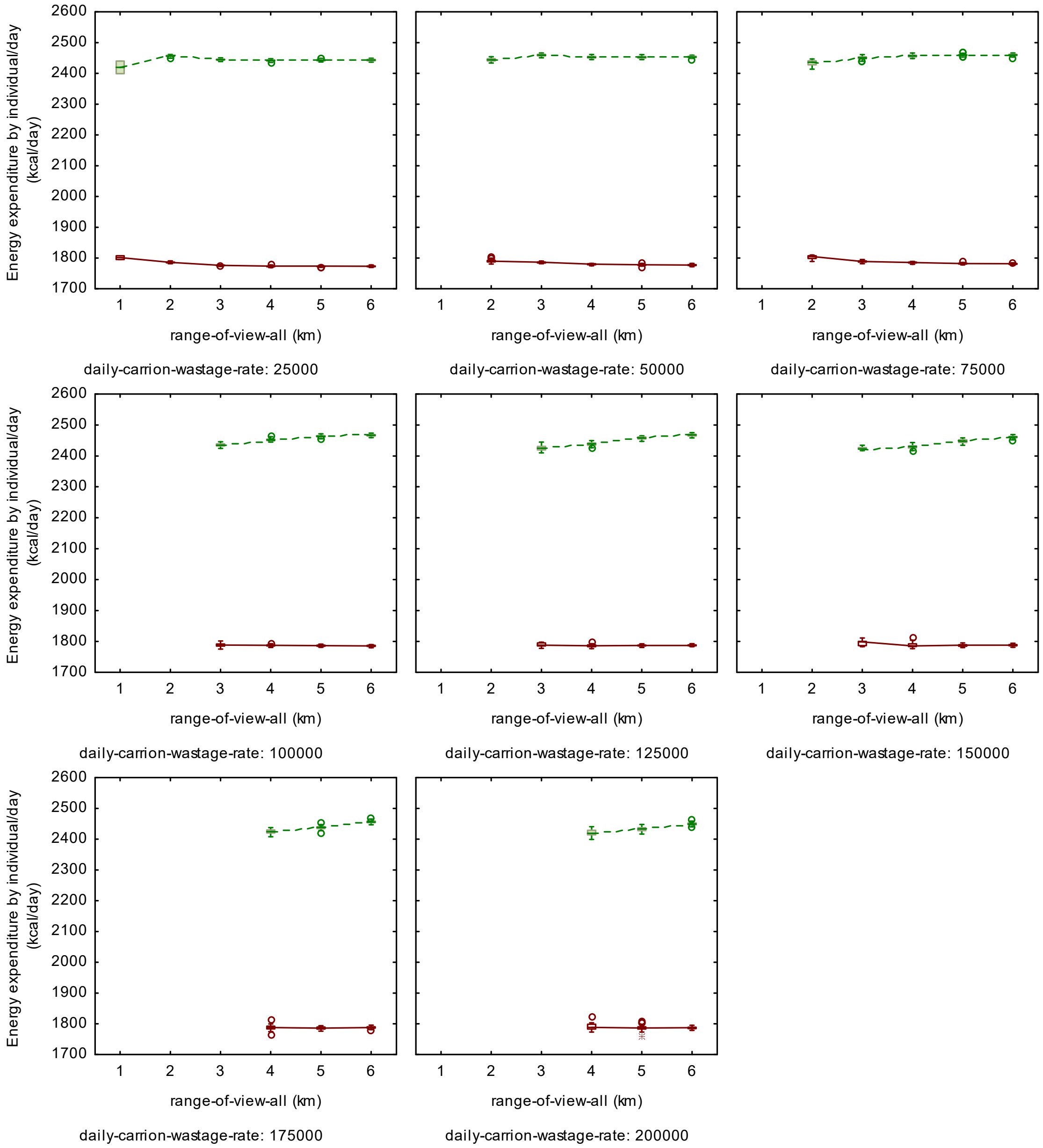

Supplementary Figure S4. Effect of range-of-view on the energetic expenditure of hominins (red line) and hyenas (green line) at different levels of daily wastage rate. The limits of the boxes correspond to the first and third quartiles, the median is shown with a horizontal line. The whiskers mark the maximum and minimum without outliers and extreme values. Outliers and extreme values are indicated with a white dot and an asterisk, respectively.

Carrion-wastage-rate was negatively correlated with the final number of hominins (Rho = -0.55; $p < 0.001$) and giant hyenas (Rho = -0.56; $p < 0.001$), as shown in Supplementary Figure S5. The energetic expenditure of hominins increased with daily-carrion-wastage-rate (Spearman's rho = 0.55 $p<0.001$) but the energetic expenditure of hyenas decreased slightly (Rho = -0.10, $p<0.001$, Figure S6).

In summary, high rates of daily carrion wastage require a large range-of-view to obtain a system able to maintain populations of scavengers (Supplementary Figure S5). A large range of view allows the scavengers to detect the patches with carrion before it is largely depleted under a high wastage rate. Consequently, we selected an intermediate value for the default level of the range-of-view (3 km) which is compatible with a relatively high wastage rate (100,000 kcal/day). Compared with a real-world landscape, a range-of-view

of 3 km is reasonable. It is not necessary to assume that the real hominins or giant hyenas were able to see a carcass 3 km away, just that they were able to detect its presence by the smell or by indirect signals like overflying vultures [42].

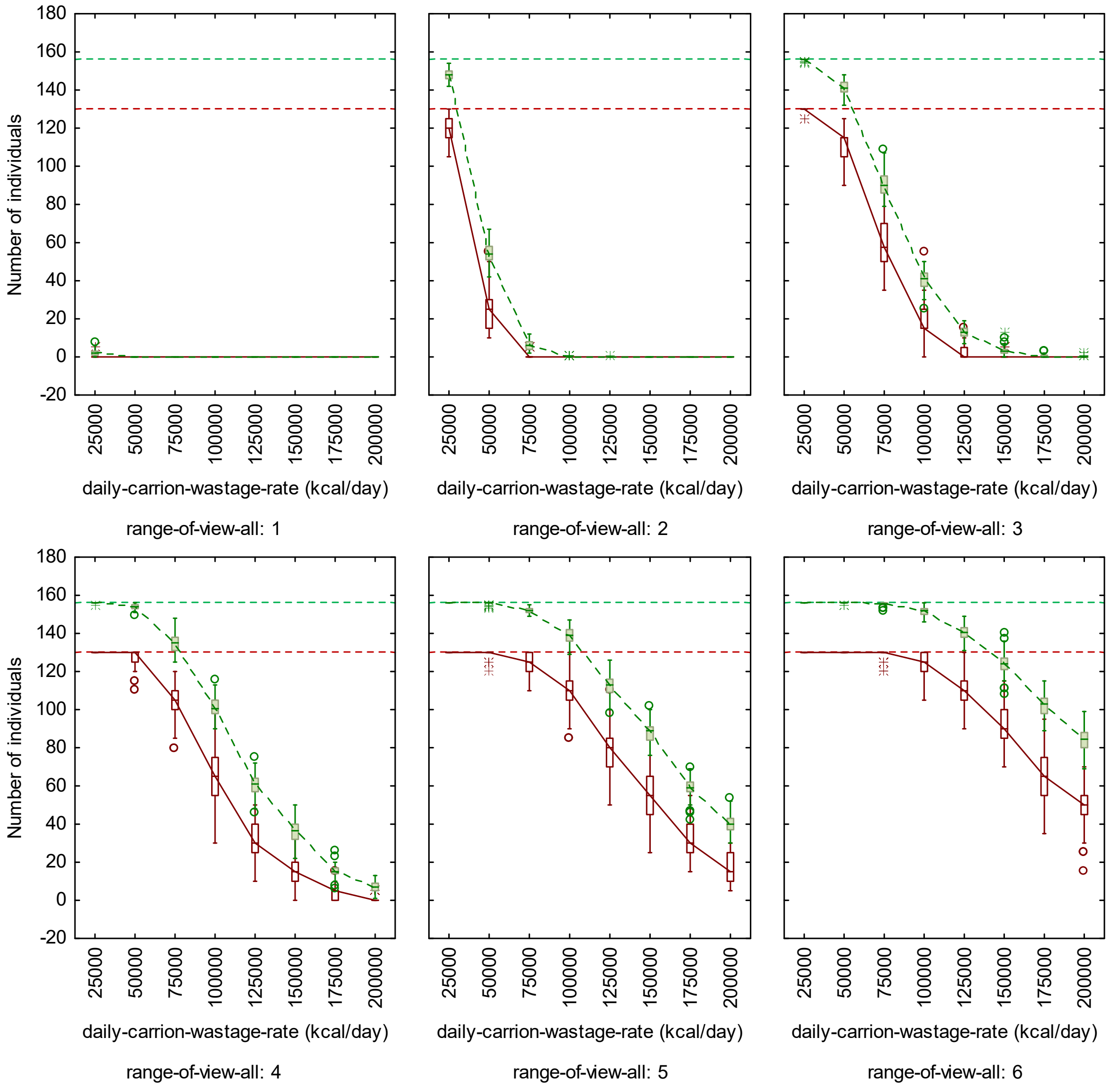

Supplementary Figure S5. Effect of daily wastage rate on the final number of hominins (red line) and hyenas (green line) at different levels of range-of-view. The initial number of hominins (red) and hyenas (green) are indicated by horizontal dashed lines. The limits of the boxes correspond to the first and third quartiles; the median is shown with a horizontal line. The whiskers mark the maximum and minimum without outliers and extreme values. Outliers and extreme values are indicated with a white dot and an asterisk, respectively.

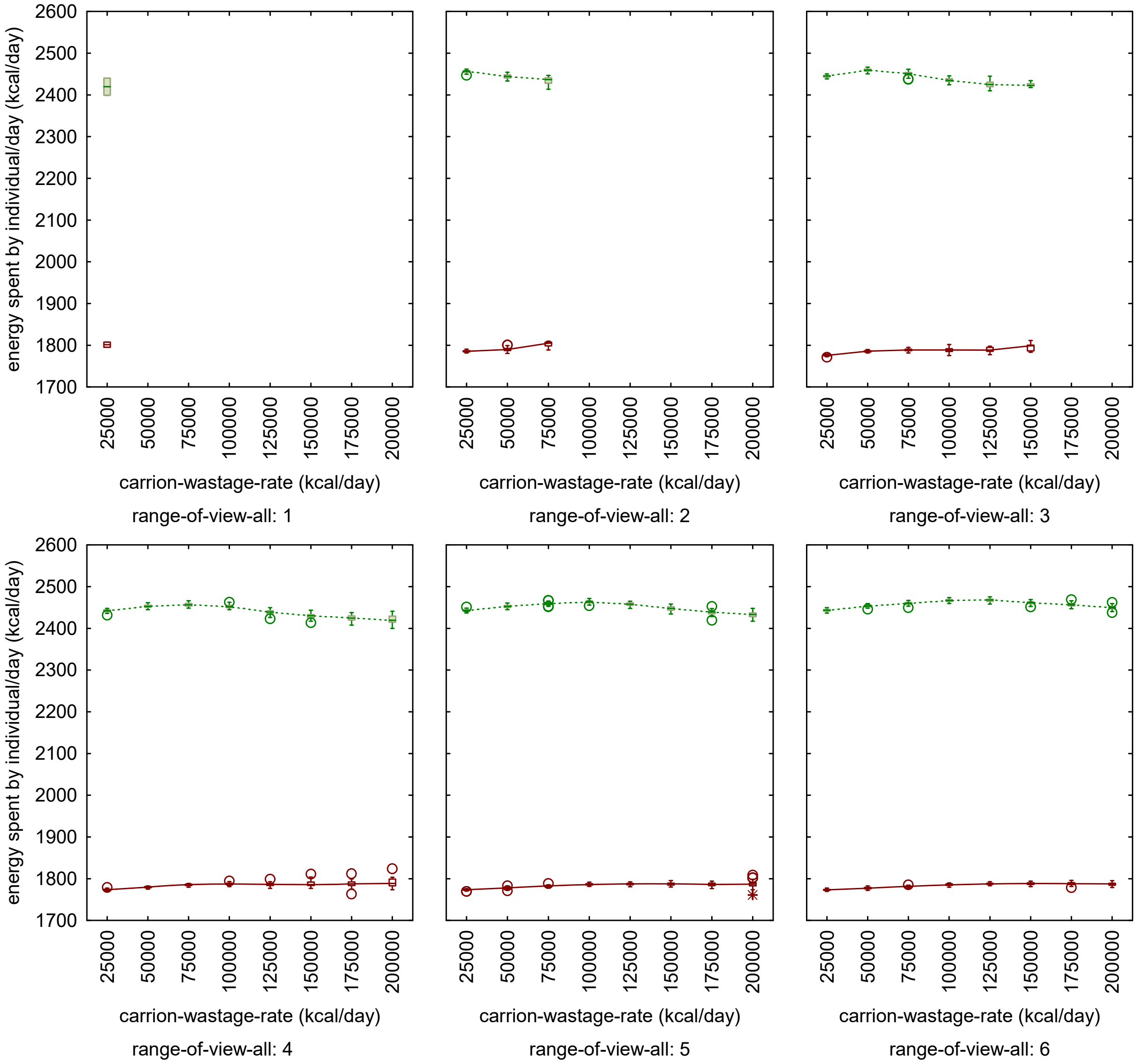

Supplementary Figure S6. Effect of daily wastage rate on the energetic expenditure of hominins (red line) and hyenas (green line) at different levels of range-of-view. The limits of the boxes correspond to the first and third quartiles; the median is shown with a horizontal line. The whiskers mark the maximum and minimum without outliers and extreme values. Outliers are indicated with a white dot.

References

1 Altolaguirre, Y., Schulz, M., Gibert, L. & Bruch, A. A. Mapping Early Pleistocene environments and the availability of plant food as a potential driver of early Homo presence in the Guadix-Baza Basin (Spain). *Journ. Hum. Evol.* **155**, 102986, doi:https://doi.org/10.1016/j.jhevol.2021.102986 (2021).

2 Rodríguez, J., Rodríguez-Gómez, G., Martín-González, J. A., Goikoetxea, I. & Mateos, A. Predator–prey relationships and the role of Homo in Early Pleistocene food webs in Southern Europe. *Palaeogeogr. Palaeoclimatol. Palaeoecol.* **365–366**, 99–114, doi:10.1016/j.palaeo.2012.09.017 (2012).

3 Espigares, M. P. *et al.* Homo vs. Pachycrocuta: Earliest evidence of competition for an elephant carcass between scavengers at Fuente Nueva-3 (Orce, Spain). *Quatern. int.* **295**, 113-125, doi:http://dx.doi.org/10.1016/j.quaint.2012.09.032 (2013).

4 Huguet, R. *et al.* Level TE9c of Sima del Elefante (Sierra de Atapuerca, Spain): A comprehensive approach. *Quatern. Int.* **433A**, 278-295, doi:http://dx.doi.org/10.1016/j.quaint.2015.11.030 (2017).

5 Blasco, R. *et al.* Earliest evidence for human consumption of tortoises in the European Early Pleistocene from Sima del Elefante, Sierra de Atapuerca, Spain. *Journ. Hum. Evol.* **61**, 503-509, doi:10.1016/j.jhevol.2011.06.002 (2011).

6 Rodríguez, J. & Mateos, A. Carrying capacity, carnivoran richness and hominin survival in Europe. *Journ. Hum. Evol.* **118**, 72-88, doi:https://doi.org/10.1016/j.jhevol.2018.01.004 (2018).

7 Lozano, S., Mateos, A. & Rodríguez, J. Exploring paleo food-webs in the European Early and Middle Pleistocene: A network analysis. *Quaternary International*, doi:http://dx.doi.org/10.1016/j.quaint.2015.10.068 (2016).

8 Moleón, M. *et al.* Humans and Scavengers: The Evolution of Interactions and Ecosystem Services. *BioScience* **64**, 394-403, doi:10.1093/biosci/biu034 (2014).

9 Altolaguirre, Y. *et al.* An environmental scenario for the earliest hominins in the Iberian Peninsula: Early Pleistocene palaeovegetation and palaeoclimate. *Review of Palaeobotany and Palynology* **260**, 51-64, doi:https://doi.org/10.1016/j.revpalbo.2018.10.008 (2019).

10 Palmqvist, P., Rodríguez-Gómez, G., Figueirido, B., García-Aguilar, J. M. & Pérez-Claros, J. A. On the ecological scenario of the first hominin dispersal out of Africa. *L'Anthropologie* **126**, 102998, doi:https://doi.org/10.1016/j.anthro.2022.102998 (2022).

11 Anton, M., Galobart, A. & Turner, A. Co-existence of scimitar-toothed cats, lions and hominins in the European Pleistocene. Implications of the post-cranial anatomy of Homotherium latidens (Owen) for comparative palaeoecology. *Quaternary Science Reviews* **24**, 1287-1301 (2005). https://doi.org/10.1016/j.quascirev.2004.09.008

12 Palmqvist, P., Grôcke, D. R., Arribas, A. & Fariña, R. A. Paleoecological reconstruction of a Lower Pleistocene large mammal community using biogeochemical ($d^{13}C$, $d^{15}N$, $d^{18}O$, Sr: Zn) and ecomorphological approaches. *Paleobiol.* **29**, 205-229 (2003). https://doi.org/10.1666/0094-8373(2003)029<0205:PROALP>2.0.CO;2

13 Palmqvist, P. *et al.* Biogeochemical and ecomorphological inferences on prey selection and resource partitioning among mammalian carnivores in an early Pleistocene community. *Palaios* **23**, 724-737 (2008). https://doi.org/10.2110/palo.2007.p07-073r

14 Antón, M. & Galobart, A. Neck function and predatory behaviour in the scimitar toothed cat *Homotherium latidens (Owen)*. *Journal of Vertebrate Paleontology* **19**, 771-784 (1999). https://doi.org/10.1080/02724634.1999.10011190

15 Antón, M. *et al.* Implications of the mastoid anatomy of larger extant felids for the evolution and predatory behaviour of sabertoothed cats (Mammalia, Carnivora,

Felidae). *Zoological Journal of the Linnean Society* **140**, 207-221 (2004). https://doi.org/10.1111/j.1096-3642.2003.00093.x
16 Turner, A. & Antón, M. *The big Cats and their fossil relatives*. (Columbia University Press, 1996).
17 Belmaker, M. *Community Structure through Time: 'Ubeidiya, a Lower Pleistocene Site as a Case Study* Ph.D. Dissertation thesis, The Hebrew University of Jerusalem., (2006).
18 Belmaker, M. Insights from carnivore community composition on the paleoecology of early Pleistocene Eurasian sites: Implications for the dispersal of hominins out of Africa. *Quatern. Int.*, doi:http://dx.doi.org/10.1016/j.quaint.2017.02.017 (2017).
19 Espigares, M. P. *et al.* The earliest cut marks of Europe: a discussion on hominin subsistence patterns in the Orce sites (Baza basin, SE Spain). *Scientific Reports* **9**, 15408, doi:10.1038/s41598-019-51957-5 (2019).
20 Courtenay, L. A. *et al.* Deciphering carnivoran competition for animal resources at the 1.46 Ma early Pleistocene site of Barranco León (Orce, Granada, Spain). *Quaternary Science Reviews* **300**, 107912, doi:https://doi.org/10.1016/j.quascirev.2022.107912 (2023).
21 Rodríguez-Gómez, G., Rodríguez, J., Martín-González, J. A. & Mateos, A. Evaluating the impact of Homo-carnivore competition in European human settlements during the early to middle Pleistocene. *Quat. Res.* **88**, 129-151, doi:10.1017/qua.2017.20 (2017).
22 Pobiner, B. L. The zooarchaeology and paleoecology of early hominin scavenging. *Evolutionary Anthropology: Issues, News, and Reviews* **29**, 68-82, doi:https://doi.org/10.1002/evan.21824 (2020).
23 DeSantis, L. R. G., Feranec, R. S., Antón, M. & Lundelius, E. L. Dietary ecology of the scimitar-toothed cat Homotherium serum. *Current Biology* **31**, 2674-2681.e2673, doi:https://doi.org/10.1016/j.cub.2021.03.061 (2021).
24 Marean, C. W. Sabertooth cats and their relevance for early hominid diet and evolution. *Journ. Hum. Evol.* **18**, 559-582 (1989). https://doi.org/10.1016/0047-2484(89)90018-3
25 Palmqvist, P. *et al.* The giant hyena Pachycrocuta brevirostris: Modelling the bone-cracking behavior of an extinct carnivore. *Quaternary International* **243**, 61-79, doi:10.1016/j.quaint.2010.12.035 (2011).
26 Domínguez-Rodrigo, M., Egeland, C. P., Cobo-Sánchez, L., Baquedano, E. & Hulbert, R. C. Sabertooth carcass consumption behavior and the dynamics of Pleistocene large carnivoran guilds. *Scientific Reports* **12**, 6045, doi:10.1038/s41598-022-09480-7 (2022).
27 Holekamp, K. E. & Kolowski, J. M. in *Handbook of the mammals of the world* Vol. 1. Carnivores (eds D. E. Wilson & R. A. Mittermeier) 234-262 (Lynx Ediciones, 2009).
28 Turner, A. & Antón, M. The giant hyaena *Pachycrocuta brevirostris* (Mammalia, Carnivora Hyaenidae). *Geobios* **29**, 455-468 (1996). https://doi.org/10.1016/S0016-6995(96)80005-2
29 Bunn, H. T. in *Meat-eating and Human Evolution.* (eds C.B. Standford & H.T Bunn) 199-218 (Oxford University Press, 2001).
30 Bickerton, D. & Szathmáry, E. Confrontational scavenging as a possible source for language and cooperation. *BMC Evolutionary Biology* **11**, 261, doi:10.1186/1471-2148-11-261 (2011).
31 Hoad, K., Robinson, S. & Davies, R. Automated selection of the number of replications for a discrete-event simulation. *JORS* **61**, 1632-1644, doi:10.1057/jors.2009.121 (2010).
32 Hölzchen, E. *et al.* Discovering the opposite shore: How did early hominins cross sea straits? *PLoS ONE* **16**, e0252885, doi:https://doi.org/10.1371/journal.pone.0252885 (2021).

33 Sunquist, M. E. & Sunquist, F. C. in *Handbook of the mammals of the world* Vol. 1. Carnivores (eds D. E. Wilson & R. A. Mittermeier) 54-169 (Lynx Ediciones, 2009).
34 Laundré, J. W. Puma energetics: a recalculartion. *The Journal of Wildlife Management* **69**, 723-732, doi:https://doi.org/10.2193/0022-541X(2005)069[0723:PEAR]2.0.CO;2 (2005).
35 Smith, J. A. *et al.* Where and when to hunt? Decomposing predation success of an ambush carnivore. *Ecology* **101**, e03172, doi:https://doi.org/10.1002/ecy.3172 (2020).
36 Pobiner, B. L. New actualistic data on the ecology and energetics of hominin scavenging opportunities. *Journ. Hum. Evol.* **80**, 1-16, doi:http://dx.doi.org/10.1016/j.jhevol.2014.06.020 (2015).
37 Wilmers, C. C., Crabtree, R. L., Smith, D. W., Murphy, K. M. & Getz, W. M. Trophic facilitation by introduced top predators: grey wolf subsidies to scavengers in Yellowstone National Park. *Journal of Animal Ecology* **72**, 909-916, doi:https://doi.org/10.1046/j.1365-2656.2003.00766.x (2003).
38 Carbone, C. *et al.* Feeding success of African wild dogs (Lycaon pictus) in the Serengeti: the effects of group size and kleptoparasitism. *Journal of Zoology* **266**, 153-161, doi:10.1017/s0952836905006710 (2005).
39 Fuller, T., Nicholls, T. H. & Kat, P. Prey and estimated food consumption of African wild dogs in Kenya. **25**, 106-110 (1995). https://hdl.handle.net/10520/EJC116973
40 Prugh, L. & Sivy, K. J. Enemies with benefits: Integrating positive and negative interactions among terrestrial carnivores. doi: https://doi.org/10.5061/dryad.n8pk0p2rv (2021).
41 Rodríguez, J., Zorrilla-Revilla, G. & Mateos, A. Does optimal foraging theory explain the behavior of the oldest human cannibals? *Journ. Hum. Evol.* **13**, 228-239 (2019). https://doi.org/10.1016/j.jhevol.2019.03.010
42 O'Connell, J. F., Hawkes, K. & Blurton Jones, N. Hadza scavenging: Implications for Plio-Pleistocene hominid subsistence. *Curr. Anthropol* **29**, 356-363 (1988). https://doi.org/10.1086/203648
